# Impact of Social Media: A Cross-sectional Survey

**DOI:** 10.1192/bjo.2025.10284

**Published:** 2025-06-20

**Authors:** Nismen Lathif, Esha Lathif

**Affiliations:** 1 Merseycare NHS Trust, Liverpool, United Kingdom; 2 Blue Coat School, Liverpool, United Kingdom

## Abstract

**Aims:** Social media has revolutionised our lives over the past two to three decades. With the advent of smartphones we see ourselves, family members, colleagues and general public whiling time away on social media platforms. In this context it would be prudent to explore the impact of social media and one’s thoughts on the future.

**Methods:** An online survey was conducted looking into areas such as use of social media influence and opinions on adverse impact on individual use and future generation was looked into. The UK adult working population was studied and 100 individuals responded.

**Results:** 94% respondents feel mental health can be adversely affected by social media; 94% respondents worry about future generation being affected negatively by social media; 50% felt adversely affected by social media; 64% felt social media influenced them. However more that 50% of respondents used social media daily up to 4 hours despite their concerns.

**Conclusion:** Regular use of social media was very common despite one’s worries about adverse impacts on mental health. The population studied was of working adults and though they used social media regularly 94% felt worries about impact of social media on future generation. Overall the use and influence of social media on working adult population sample was high despite their own conviction that this may adversely impact mental health and future generation.

An Afterthought; Should adults model less use of social media and phone to lead the way for future generation?

